# Multi-Targeted Anti-Cancer Effects of Triptophenolide in Hormone-Responsive and Triple-Negative Breast Cancer Models

**DOI:** 10.3390/ijms26125469

**Published:** 2025-06-07

**Authors:** Zufa Sabeel, Guangshuai Chai, Ruolan Chen, Lu Ying, Yan Liu, Wenjing Zhang, Shangyang Pan, Xiaoyang Chen, Changyuan Yu, Zhao Yang

**Affiliations:** State Key Laboratory of Green Biomanufacturing, College of Life Science and Technology, Innovation Center of Molecular Diagnostics, Beijing University of Chemical Technology, Beijing 100029, China; zufasabeel183@gmail.com (Z.S.); chaiguangshuai24@mails.ucas.ac.cn (G.C.); chen13120171322@163.com (R.C.); yl_tlm@163.com (L.Y.); liuy1993@taru.edu.cn (Y.L.); zhangwenjing0818@163.com (W.Z.); 2024211052@buct.edu.cn (S.P.); chenxiaoyang0225@163.com (X.C.)

**Keywords:** breast cancer, proliferation, triptophenolide, apoptosis, cell cycle arrest, xenograft tumor model, natural product

## Abstract

Breast cancer (BC) remains a significant therapeutic challenge, necessitating novel agents with multi-target efficacy. Here, we demonstrate that triptophenolide (TRI), a bioactive compound from Tripterygium wilfordii, exerts potent anti-BC activity across hormone-responsive (MCF-7) and triple-negative (MDA-MB-231) subtypes. In vitro, TRI inhibited proliferation in a concentration-dependent manner, with IC_50_ values decreasing from 180.3 μg/mL (24 h) to 127.2 μg/mL (48 h) in MCF-7 cells, and from 322.5 μg/mL to 262.1 μg/mL in MDA-MB-231 cells. TRI treatment induced G1-phase arrest in both breast cancer subtypes, increasing the G1 population by 22.27% in MCF-7 cells and 10.64% in MDA-MB-231 cells. Concurrently, TRI triggered apoptosis, elevating apoptotic rates from 3.36% to 9.78% in MCF-7 cells and from 7.01% to 17.02% in MDA-MB-231 cells. These effects were associated with the significant upregulation of pro-apoptotic proteins BAX, BAK1, BIM, and cytochrome c (CYCS). Notably, TRI suppressed migration by 61.5% (MCF-7) and 71.5% (MDA-MB-231). In vivo, TRI treatment inhibited MCF-7 xenograft growth and reduced tumor volume (1207.5 vs. 285 mm^3^) and weight (0.22 vs. 0.1 g), while extending the survival time of tumor-bearing mice from 14–20 days to 24 days. These results position TRI as a promising lead therapeutic candidate against diverse BC subtypes, with mechanistic versatility surpassing single-target agents.

## 1. Introduction

Breast cancer (BC) has become the most commonly diagnosed malignancy worldwide, representing 31% of all female cancer cases. Projections suggest a 40% increase in incidence by 2040 [1]. Monitoring this growing burden is crucial for evaluating cancer control initiatives, including the WHO Global Breast Cancer Initiative’s target of 2.5% annual mortality reduction. GLOBOCAN 2022 data reported 2.3 million new cases and 670,000 deaths globally in 2022, with comprehensive projections through 2050 across 185 countries [2]. Despite improved survival rates, BC continues to impose a substantial disease burden and remains a leading cause of mortality among women aged 30–60 in China, highlighting the urgent need for improved treatment strategies [3].

BC’s heterogeneity, categorized into Luminal A, Luminal B, HER2-enriched, and triple-negative subtypes, complicates treatment selection [4,5]. Endocrine therapies like tamoxifen have improved outcomes, but intrinsic or acquired resistance develops in 20–30% of patients, limiting long-term efficacy [6]. Current modalities—surgery, chemotherapy, radiotherapy, and targeted therapy—often cause severe side effects, including hepatotoxicity and reduced quality of life [7,8,9,10,11,12].

Traditional Chinese Medicine (TCM) has garnered attention for its multi-modal anti-tumor effects and historical efficacy in BC management [13]. TCM theory links BC pathogenesis to emotional stress and dietary factors, disrupting the Chong and Ren meridians [14,15]. Preclinical studies validate TCM-derived compounds (e.g., pseudolaric acid B, carnosic acid) for their ability to inhibit proliferation, induce apoptosis, and synergize with conventional therapies [14,15]. Our group identified glabridin, ligustilide, paeonol, and medicarpin as promising candidates against bladder cancer and glioblastoma [16,17,18,19], supporting TCM’s potential in precision oncology. Natural compounds such as quercetin, tetrandrine, thymoquinone, resveratrol, honokiol, garcinol, biochanin A, lycopene, shikonin, sulforaphane, and carotenoids have been shown to inhibit BC by inducing apoptosis, cell cycle arrest, and autophagy, while exerting minimal toxicity on normal cells [20]. Their low toxicity and cancer-specific actions make them promising candidates for BC therapy [21]. Ongoing research may expand their clinical use and improve survival outcomes.

Triptophenolide (TRI), a bioactive diterpenoid from Tripterygium wilfordii (TW), exhibits anti-tumor, anti-inflammatory, and immunomodulatory properties [22,23]. TW-based drugs (e.g., Tripterygium Glycosides Tablets) are clinically approved in China for autoimmune diseases [24], while TW extracts demonstrate efficacy against liver, lung, and hematologic malignancies [25,26,27,28,29,30]. However, TRI’s mechanisms remain poorly characterized compared to other TW constituents like triptolide [31]. A recent study identified TRI-14-O-β-D-glucopyranoside, a cytotoxic derivative active against BC (MCF-7), glioblastoma, and leukemia cells [32], suggesting TRI’s untapped therapeutic value.

Currently, preliminary studies have shown that TRI possesses the ability to suppress acute inflammation in mice [32] and can modulate T lymphocyte-mediated immune responses. While TW is known to contain several well-characterized bioactive molecules, such as triptolide [33] and tripterine [31,34], the pharmacological properties of TRI remain relatively underexplored [22]. Notably, unlike triptolide, which exhibits potent anti-cancer activity [33] but is highly toxic [35,36], TRI demonstrates moderate cytotoxicity and a potentially more favorable safety profile [36], making it a promising alternative for further development. Moreover, TRI’s immune-modulatory effects appear mechanistically distinct, as suggested by early findings on T-cell regulation. These aspects collectively underscore TRI’s pharmacological novelty and justify its further investigation as a potential therapeutic agent [22,37].

This study pioneers the investigation of pure TRI monomer in BC, elucidating its molecular targets and mechanisms. By addressing this gap, we aim to advance TRI’s translational potential as a complementary or alternative therapy, offering a safer, multi-targeted approach to BC management.

## 2. Results

### 2.1. TRI Inhibits BC Cell Proliferation in a ConcentrationDependent Manner

**Figure 1:**
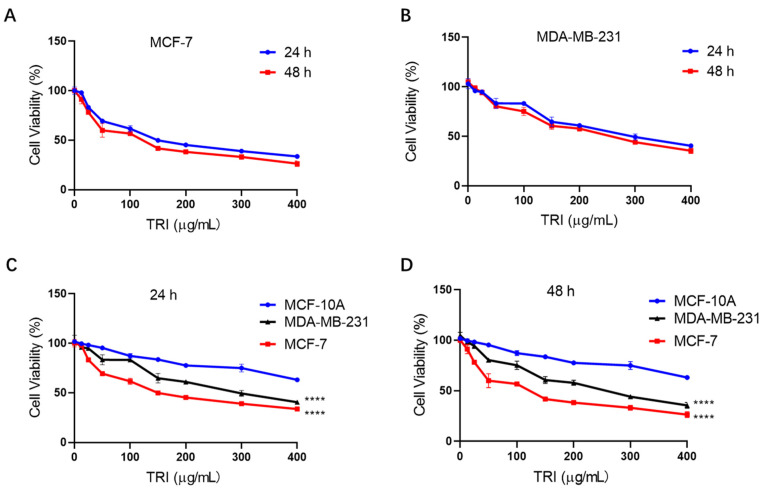
Effect of TRI (0–400 µg/mL) on cell proliferation in BC (MCF-7 and MDA-MB-231) and normal breast cells (MCF-10A) assessed by CCK-8 assay. (**A**) MCF-7 cells treated for 24 h and 48 h. (**B**) MDA-MB-231 cells treated for 24 h and 48 h. (**C**) Comparative analysis of MCF-7, MDA-MB-231, and MCF-10A cells after 24 h treatment. (**D**) Comparative analysis of MCF-7, MDA-MB-231, and MCF-10A cells after 48 h treatment. Data are presented as mean ± SEM; **** *p* < 0.0001.

Cell proliferation assays revealed potent anti-tumor activity of TRI against BC cells. Treatment with TRI (0–400 μg/mL) for 24 h yielded IC_50_ values of 180.3 μg/mL (MCF-7) and 322.5 μg/mL (MDA-MB-231), while 48 h exposure resulted in significantly lower IC_50_ values of 127.2 μg/mL and 262.1 μg/mL, respectively (Figure 1A,B).

Notably, TRI exhibited selective cytotoxicity against malignant cells. At the highest concentration tested (400 μg/mL), TRI caused significantly greater reduction in viability of BC cells (MCF-7 and MDA-MB-231) after both 24 h (*p* < 0.0001) and 48 h (*p* < 0.0001) treatments (Figure 1A,B). Compared with normal breast cells (MCF-10A), BC cells (MCF-7 and MDA-MB-231) treated with 0–400 μg/mL TRI for 24 h showed a significant decrease in cell viability (*p* < 0.0001) (Figure 1C), and the same treatment resulted in a similarly significant decrease in cell viability for 48 h (*p* < 0.0001) (Figure 1D). Significance indicators denote overall differences between cell types across the dose range rather than at individual concentrations (Figure 1C,D). Collectively, the anti-proliferative effects of TRI showed clear concentration dependence across all tested cell lines. These findings demonstrate that TRI preferentially inhibits BC cell proliferation while showing reduced cytotoxicity toward normal cells, suggesting a favorable therapeutic window for further development.

### 2.2. TRI Induces Cell Cycle Arrest in BC Cells

**Figure 2:**
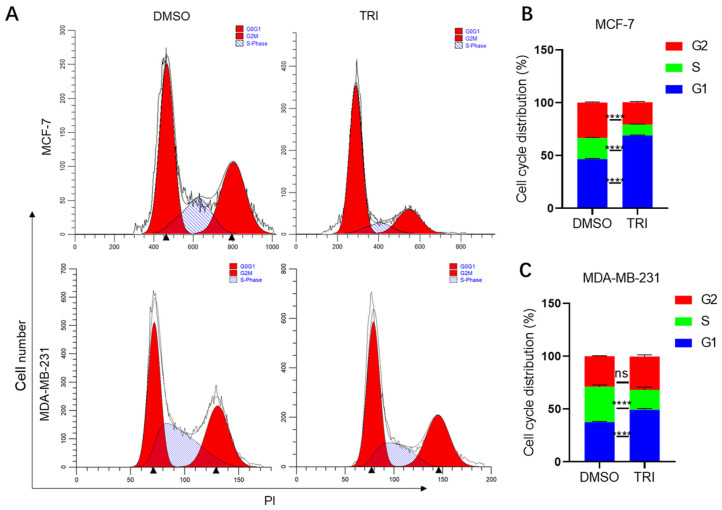
Cell cycle analysis of BC cells (MCF-7 and MDA-MB-231) treated with TRI for 48 h by flow cytometry. (**A**) Cell cycle distribution profiles of MCF-7 and MDA-MB-231 cells. (**B**) Histogram representation of MCF-7 cell cycle phases. (**C**) Histogram representation of MDA-MB-231 cell cycle phases. Data are presented as mean ± SEM; **** *p* < 0.0001.

The cell cycle represents a fundamental biological process governing cellular proliferation and homeostasis. In malignant transformation, dysregulation of cell cycle checkpoints frequently occurs, leading to uncontrolled tumor growth and proliferation [38]. Flow cytometric analysis demonstrated that 48 h TRI treatment (150 μg/mL for MCF-7; 290 μg/mL for MDA-MB-231) significantly increased G1 phase populations in both BC cell lines (Figure 2A). MCF-7 cells showed a 22.27% increase in G1 phase accumulation (46.08% to 68.35%, *p* < 0.0001; Figure 2A,B), while MDA-MB-231 cells exhibited a 10.64% increase (37.37% to 48.01%, *p* < 0.0001; Figure 2A,C). ModiFit analysis confirmed these G1 arrest patterns, suggesting TRI may target G1 checkpoint regulators, potentially through cyclin-dependent kinase modulation. The observed dose-dependent effects (Figure 2C) further support TRI’s specific activity against cell cycle progression in malignant cells. These findings demonstrate TRI’s ability to disrupt fundamental cell cycle progression in BC cells.

### 2.3. TRI Induces Apoptosis in BC Cells

**Figure 3:**
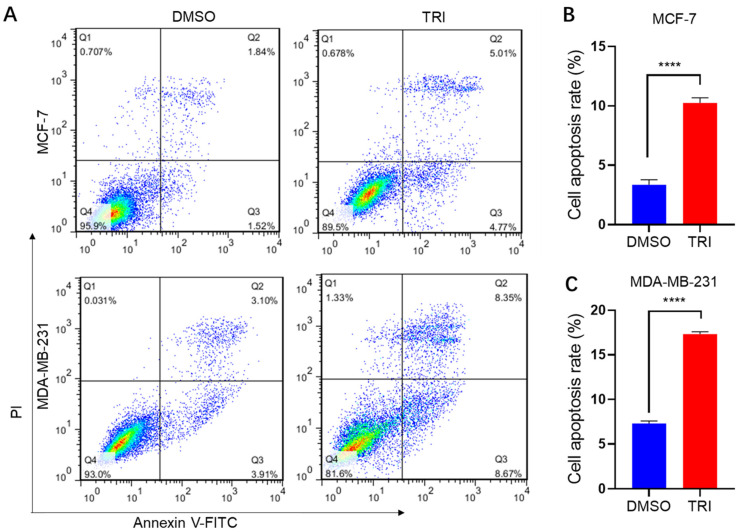
Apoptotic effects of TRI on breast cancer (BC) cells (MCF-7 and MDA-MB-231) after 48 h treatment assessed by flow cytometry. (**A**) Apoptosis profiles (early/late apoptosis and necrosis) of MCF-7 and MDA-MB-231 cells. (**B**) Quantitative histogram of apoptosis in MCF-7 cells. (**C**) Quantitative histogram of apoptosis in MDA-MB-231 cells. Data represent mean ± SD; **** *p* < 0.0001.

The anti-proliferative effect of TRI on BC cells was first confirmed by in vitro cell viability assays. To further elucidate TRI’s mechanism of action, we assessed its ability to induce apoptosis using Annexin V-FITC/PI staining followed by flow cytometric analysis. MCF-7 and MDA-MB-231 BC cells were treated with 100 μg/mL and 250 μg/mL TRI, respectively, for 24 h. Flow cytometric analysis revealed a dose-dependent increase in apoptosis across both cell lines (Figure 3A). In MCF-7 cells, TRI treatment (100 μg/mL) elevated the total apoptotic population (early + late apoptosis) from 3.36% to 9.78% (*p* < 0.0001) (Figure 3A,B). Similarly, MDA-MB-231 cells exhibited a pronounced apoptotic response, with the apoptotic fraction increasing from 7.01% to 17.02% following 250 μg/mL TRI exposure (*p* < 0.0001) (Figure 3A,C). These data demonstrate a 2–3-fold induction of apoptosis, underscoring TRI’s potent anti-tumor activity.

The differential distribution of cell populations across quadrants further validated TRI’s mechanistic action: early apoptotic cells (Annexin V^+^/PI^−^) predominated in quadrant Q4, whereas late apoptotic cells (Annexin V^+^/PI^+^) localized to quadrant Q2. Notably, the shift in apoptotic indices correlated with reduced viability (Q3: Annexin V^−^/PI^−^), suggesting TRI-mediated cytotoxicity. Collectively, these findings establish TRI as a promising apoptosis-inducing agent in BC models, warranting further investigation into its molecular targets and therapeutic potential.

### 2.4. TRI Suppresses BC Cell Migration

**Figure 4:**
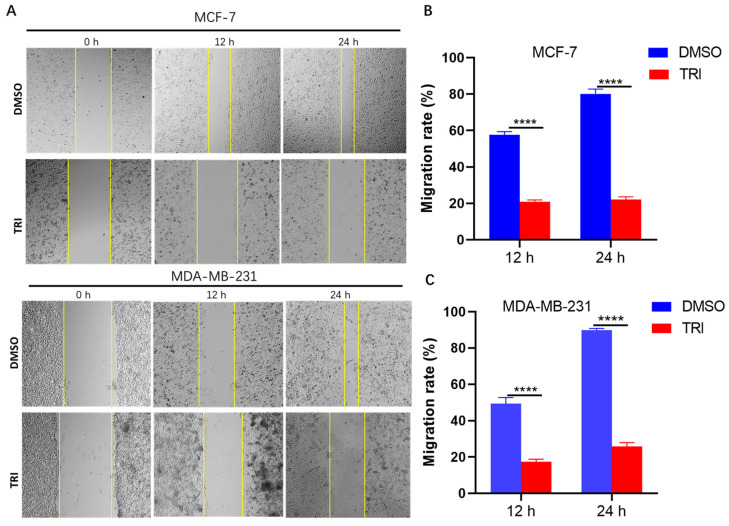
Cell migration of breast cancer (BC) cells treated with TRI at 0 h, 12 h, and 24 h assessed by wound healing assay. (**A**) Representative migration images of MCF-7 and MDA-MB-231 cells. (**B**) Quantification of cell migration in MCF-7 cells. (**C**) Quantification of cell migration in MDA-MB-231 cells. Data are presented as mean ± SEM; **** *p* < 0.0001.

Cell migration represents a fundamental characteristic of malignant tumors, playing a pivotal role in cancer progression and metastasis. To investigate the inhibitory effects of TRI on BC cell motility, we conducted wound healing assays using two distinct BC cell lines: MCF-7 and MDA-MB-231. TRI significantly inhibits BC cell migration, as demonstrated by wound healing assays. Treatment with TRI for 12 h markedly reduced the migration rate of MCF-7 cells from 57.5% to 20.5% (*p* < 0.0001) and MDA-MB-231 cells from 52% to 18.5% (*p* < 0.0001) (Figure 4A,B). After 24 h of exposure, the inhibitory effect was even more pronounced: MCF-7 migration decreased sharply from 83% to 23.5% (*p* < 0.0001), while MDA-MB-231 migration dropped from 90% to 28.5% (*p* < 0.0001) (Figure 4A,C). These findings clearly indicate that TRI exerts a potent, time-dependent suppression of BC cell motility, highlighting its potential as an anti-migratory therapeutic agent.

### 2.5. TRI Induces Apoptosis in BC Cells by Modulating the Expression of Key Apoptotic Genes

**Figure 5:**
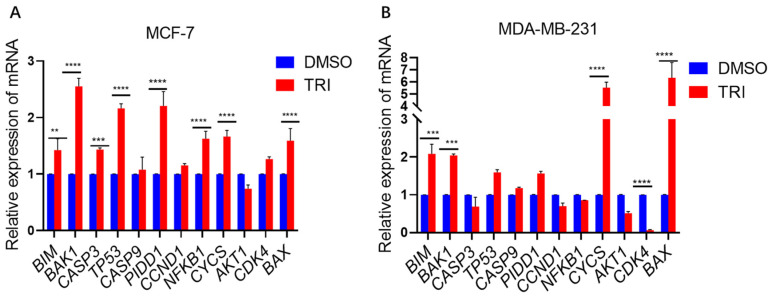
qRT-PCR analysis of gene expression in MCF-7 and MDA-MB-231 cells following TRI treatment. (**A**) Relative mRNA expression levels in MCF-7 cells. (**B**) Relative mRNA expression levels in MDA-MB-231 cells. Data are presented as mean ± SD (*n* = 3 biological replicates); ** *p* < 0.01, *** *p* < 0.001, **** *p* < 0.0001.

TRI significantly modulated apoptosis-related gene expression in BC cells as determined by SYBR Green-based qPCR. Following 48 h of TRI treatment at concentrations equivalent to those used in cell cycle experiments, we observed distinct patterns of apoptotic gene regulation in MCF-7 and MDA-MB-231 cell lines (Figure 5). In MCF-7 cells, TRI treatment induced significant upregulation (*p* < 0.05) of pro-apoptotic genes including *BAK1* (2.65-fold), *BIM* (1.57-fold) (*p* < 0.01), *CASP3* (1.53-fold) (*p* < 0.001), and *TP53* (2.3-fold) (*p* < 0.0001), along with *PIDD1* (2.26-fold) (*p* < 0.0001), *NFKB1* (1.7-fold) (*p* < 0.0001), *CYCS* (1.8-fold) (*p* < 0.0001), and *BAX* (1.6-fold) (*p* < 0.0001) (Figure 5A). The MDA-MB-231 cell line similarly showed marked elevation in *BIM* (2.2-fold) (*p* < 0.001), *BAK1* (2.22-fold) (*p* < 0.001), *CYCS* (5.9-fold) (*p* < 0.0001), and *BAX* (6.9-fold) expression (*p* < 0.0001). Notably, *CDK4* expression was significantly suppressed (0.1-fold decrease, *p* < 0.01) in MDA-MB-231 cells (Figure 5B), consistent with TRI’s observed cell cycle inhibitory effects. Gene expression quantification was performed using the 2^−ΔΔCt^ method with GAPDH normalization. These findings demonstrate that TRI promotes apoptosis in BC cells through coordinated regulation of both intrinsic apoptotic pathway components and cell cycle regulators, with particularly strong effects on executioner caspases (CASP3) and Bcl-2 family proteins (BAX, BAK1).

### 2.6. WB Analysis of Apoptosis-Related Protein Expression in Response to TRI Treatment

**Figure 6:**
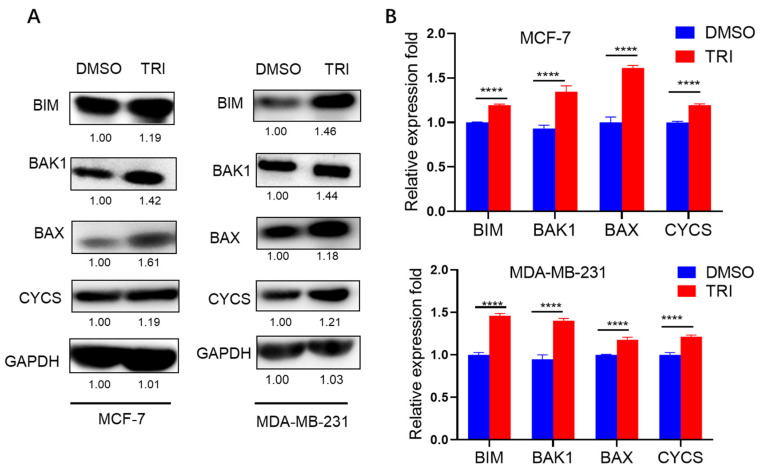
Molecular mechanism of TRI-induced apoptosis in breast cancer cells (MCF-7 and MDA-MB-231) after 48 h treatment. (**A**) Western blot analysis of apoptosis-related proteins in MCF-7 and MDA-MB-231 cells, with GAPDH as loading control. Top: representative blots; bottom: quantified protein expression levels (relative to GAPDH). (**B**) Histogram showing relative protein expression levels from three independent experiments. Data presented as mean ± SD (*n* = 3); **** *p* < 0.0001.

WB analysis confirmed the protein-level regulation of key apoptotic markers identified through RT-qPCR screening (Figure 6). Four pro-apoptotic proteins—BIM, BAK1, BAX, and CYCS—were selected for validation based on their consistent mRNA upregulation patterns in both MCF-7 and MDA-MB-231 cell lines. Quantitative analysis demonstrated significant TRI-induced upregulation of all four targets (BIM, BAK1, BAX, and CYCS; *p* < 0.0001) compared to the control group. These protein expression patterns showed strong correlation with mRNA levels (R^2^ = 0.89), confirming TRI’s ability to coordinately regulate both transcriptional and translational components of the apoptotic cascade. The consistent upregulation of these BCL-2 family proteins and cytochrome c provides compelling evidence for TRI’s activation of the intrinsic apoptosis pathway in BC cells.

### 2.7. TRI-Induced Suppression of Tumor Progression in a Nude Mouse Model

**Figure 7:**
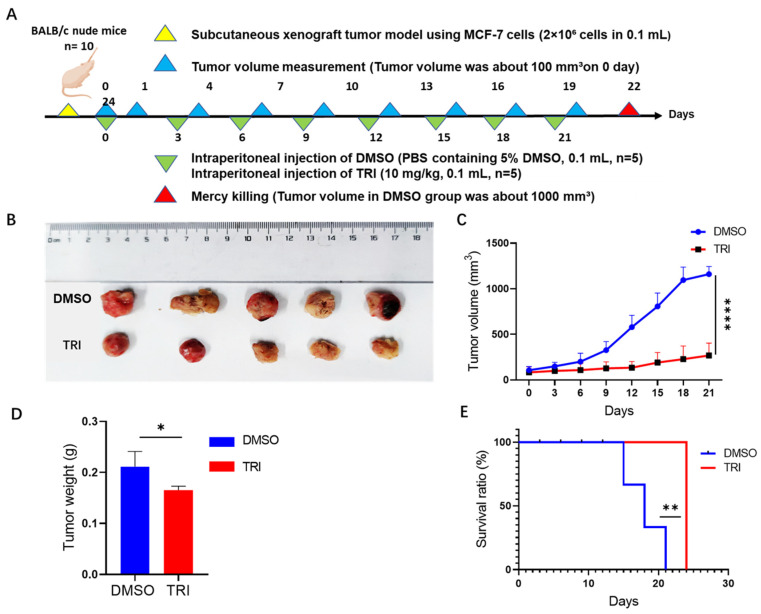
Xenograft tumor model. (**A**) Experimental timeline showing tumor inoculation, measurement, and drug treatment in nude mice. (**B**) Representative images of tumors from euthanized mice treated with TRI (TRI inhibitor) or DMSO (control). (**C**) Tumor volume measurements in mice treated with TRI vs. DMSO. (**D**) Tumor weight in mice treated with TRI vs. DMSO. (**E**) Survival analysis of mice treated with TRI vs. DMSO. * *p* < 0.05, ** *p* < 0.01, **** *p* < 0.0001.

Our in vitro studies (Figure 1, Figure 2, Figure 3, Figure 4, Figure 5 and Figure 6) demonstrated TRI’s inhibitory effect on BC (MCF-7 and MDA-MB-231). To further evaluate TRI’s efficacy in vivo, we established a xenograft tumor model in nude mice (Figure 7A). Tumor volumes in the TRI-treated group were consistently smaller than those in the DMSO control group (Figure 7B,C). Compared to controls, TRI significantly reduced tumor volume (from 1207.5 mm^3^ to 285 mm^3^; *p* < 0.0001; Figure 7C) and weight (from 0.22 g to 0.1 g; *p* < 0.05; Figure 7D), while also improving survival rates (day 24 vs. control deaths at days 14–20; *p* < 0.01; Figure 7E). These results confirm that TRI suppresses BC (MCF-7) tumorigenesis in vivo and prolongs survival in tumor-bearing mice.

**Figure 8:**
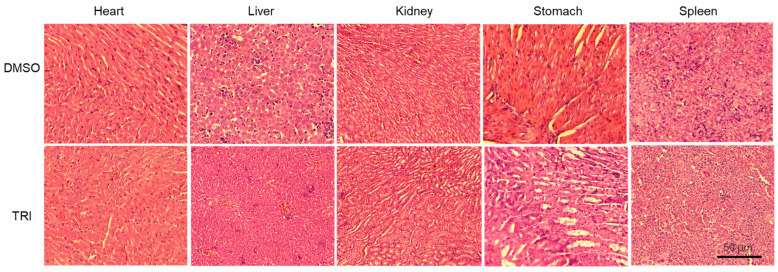
H&E-stained sections of major organs (heart, liver, kidney, stomach, and spleen) from control and TRI-treated mice in the BC xenograft model. No significant histopathological changes were observed in TRI-treated mice, indicating minimal systemic toxicity. Scale bar: 50 µm.

Histopathological evaluation revealed no lesions or abnormalities in the organs of either TRI-treated or control mice (Figure 8), indicating that TRI is non-toxic and well tolerated in vivo. Together, our findings validate TRI’s potent anti-tumor effects in both in vitro and in vivo models.

### 2.8. Histopathological Evaluation of Major Organs

To assess the potential toxicity of TRI, major organs including the heart, liver, kidney, stomach, and spleen were collected from control and TRI-treated mice and analyzed using H&E staining. As shown in Figure 8, no significant histological abnormalities were observed in the TRI-treated group compared to controls. Only mild, non-specific changes were noted in some organs, indicating that TRI treatment does not cause overt tissue damage at the tested dose.

## 3. Discussion

Nowadays, it is understood that cancer is a growing ecosystem in which cancer cells successfully communicate with their surroundings. Because of tumor heterogeneity, resistance to chemotherapeutic drugs used to treat cancer in general and BC in particular is growing, which has made the development of novel medicines necessary [39]. Compounds with an OH moiety have been shown to have several intriguing features; anti-cancer activity is one of these properties that has recently attracted more attention [40,41]. Currently, the world’s research on natural chemicals with anti-cancer properties is looking promising.

Plant-derived, herbal, and marine natural products have emerged as promising anti-cancer agents, with their bioactive constituents demonstrating the ability to modulate critical cellular pathways, especially apoptosis [42]. While conventional BC treatments remain effective, they face significant limitations. TCM has emerged as a valuable complementary approach in cancer prevention and therapy. With its multi-target therapeutic capabilities, TCM shows growing promise in BC treatment, demonstrating efficacy against both tumor growth and chemotherapy/radiotherapy side effects [43].

For over two millennia, TW has been used in traditional medicine to treat various conditions including parasitic infections, inflammatory swellings, and breast abscesses. Centuries of research have refined TW into clinically viable botanical extracts. Modern clinical trials have validated its efficacy in treating autoimmune disorders (rheumatoid arthritis, ankylosing spondylitis, SLE) and dermatological conditions, and in managing post-transplant care [44].

TRI, a non-glycosidic small molecule derived from TW, has been implicated in anti-inflammatory and anti-tumor activities [45]. Our study provides the first demonstration of its significant anti-tumor efficacy against BC. While these findings are promising, the precise regulatory mechanisms mediating TRI’s effects on BC remain incompletely understood. To address this knowledge gap, we conducted comprehensive in vitro and in vivo investigations, which revealed that TRI likely mediates its anti-cancer effects through the induction of apoptosis. TRI treatment significantly inhibited proliferation, induced cell cycle arrest, triggered apoptosis, and suppressed migration in both MCF-7 and MDA-MB-231 BC cell lines, demonstrating multi-modal anti-tumor activity across molecular subtypes (Figure 1, Figure 2, Figure 3, Figure 4, Figure 5, Figure 6, Figure 7 and Figure 8). While the current study employed MCF-7 and MDA-MB-231 cell lines to represent ER-positive and triple-negative breast cancer subtypes, respectively, future research should expand the panel of cell lines to include HER2-positive models such as SK-BR-3. This will allow for a more comprehensive evaluation of TRI’s anti-cancer potential across diverse molecular subtypes and help determine its broader clinical applicability.

Dysregulated cell proliferation serves as a fundamental driver of oncogenesis and tumor development [46]. Our study demonstrates that TRI exhibits time-dependent cytotoxicity in BC cells, with significantly enhanced potency after 48 h of exposure compared to 24 h (IC_50_ values decreasing from 180.3 to 127.2 μg/mL in MCF-7 and from 322.5 to 261.1 μg/mL in MDA-MB-231 cells). This pattern of cumulative anti-cancer activity aligns with observations for other bioactive herbal compounds, including thymol/carvacrol from *Thymus vulgaris* (direct cytotoxicity), *Hibiscus* polyphenols (proliferation inhibition), artemisinin from *Artemisia annua* (cell cycle and angiogenesis modulation), and quercetin/kaempferol from *Moringa oleifera* (metastasis suppression via apoptosis) [47]. The greater sensitivity of hormone-responsive MCF-7 cells compared to triple-negative MDA-MB-231 cells suggests potential receptor-mediated mechanisms that warrant further investigation. Although TRI exhibited promising anti-cancer activity at sub-cytotoxic concentrations, its relatively high IC_50_ values raise concerns about potential off-target effects. Future studies should focus on identifying precise molecular targets and optimizing delivery strategies to enhance TRI’s translational potential.

The cell cycle is a tightly regulated process whose dysregulation drives tumorigenesis [46]. In TRI-treated BC cells, we observed G1 phase arrest (Figure 2), though the underlying molecular mechanisms remain to be elucidated. Cell cycle progression is primarily governed by cyclin-dependent kinases and their regulatory proteins, with two critical checkpoints: G1/S and G2/M [19]. Notably, cancer cells typically exhibit G1/S checkpoint impairment while maintaining functional G2/M checkpoint control, which serves as a crucial survival mechanism for malignant cells [48]. These findings suggest that TRI exerts its anti-cancer effects primarily through G1 phase arrest, distinguishing it from therapeutic agents that target S or G2/M phase cell cycle checkpoints. The present study identifies TRI as a novel cell cycle-modulating agent derived from TCM, demonstrating specific G1-phase arrest in BC cells. This effect mirrors the established activity of other TCM phytochemicals (e.g., quercetin) [49] while exhibiting distinct pharmacological properties. These findings establish TRI as a promising TCM-derived agent with specific cell cycle-targeting activity against BC, similar to other bioactive phytochemicals. However, a limitation of this study is the lack of investigation into TRI’s effects on normal breast epithelial cells (MCF-10A), which is crucial for assessing its selectivity and potential toxicity. Future studies should include normal cell line models to evaluate TRI’s safety profile and confirm its selective anti-cancer effects, which would be essential for its clinical translation.

Apoptosis is a programmed cell death mechanism characterized by orderly cellular elimination. This process is executed through caspase activation, initiated via either the extrinsic (death receptor) or intrinsic (mitochondrial) pathways [17]. In this study, we investigated TRI-induced apoptosis in BC cells and its effects on key apoptotic proteins (BIM, BAK1, BAX, and CYCS). Our results demonstrate that TRI treatment significantly increased apoptosis rates in both BC cell lines, from 3.36% to 9.78% in MCF-7 cells (Figure 3A,B) and from 7.01% to 17.02% in MDA-MB-231 cells (Figure 3A,C). WB analyses consistently revealed upregulation of key intrinsic apoptotic pathway components (BIM, BAK1, BAX, CYCS) across both cell types. While qPCR data showed cell line-specific variations in CASP3 and PIDD1 expression, the overall protein profile confirms TRI’s activation of mitochondrial-mediated apoptosis. This mechanism shares features with other TCM-derived compounds like kuwanon C (which promotes Bax oligomerization) [50] and rhoifolin (known to disrupt mitochondrial function) [51]. However, the cell line-dependent differences in CASP3 and PIDD1 expression suggest that TRI induces apoptosis via distinct pathways: synergizing intrinsic and extrinsic signaling in MCF-7 cells and primarily activating mitochondrial pathways in MDA-MB-231 cells. This highlights TRI as a multi-target agent with subtype-specific mechanisms. A limitation of this study is the focus on BCL-2 family proteins without comprehensive analysis of downstream apoptotic effectors. While CASP3 was significantly regulated in MCF-7 cells, it was undetectable in MDA-MB-231 cells, and CASP9 expression was non-significant in both lines, precluding protein-level validation of caspase activation and PARP cleavage. Future work should include detailed assessment of caspase cascade activation to fully elucidate TRI’s apoptotic mechanisms.

Metastasis represents a major cause of therapeutic resistance and treatment failure in BC [52]. The current study reveals that TRI significantly inhibits BC cell migration, underscoring its anti-metastatic potential. This aligns with other TCM agents like curcumin, known to suppress BC invasion [53]. The STAT3 signaling pathway has been well-characterized as a critical driver of cancer metastasis through its regulation of matrix metalloproteinases (MMPs) [54]. While our findings suggest that TRI inhibits BC cell migration, the involvement of the STAT3-MMP axis remains hypothetical, as we did not directly assess STAT3 activation or MMP expression. Future studies should experimentally validate this mechanism by evaluating STAT3 phosphorylation, MMP-2/9 activity, and EMT markers. Clarifying TRI’s role in this pathway may align it with other TCM-derived agents known to target STAT3 signaling [52].

In a subcutaneous BC mouse model, TRI significantly inhibited tumor growth and prolonged survival (Figure 7). Histopathological analysis, as shown in Figure 8, revealed no significant abnormalities and only mild changes, if any, in major organs following TRI treatment. These findings suggest that TRI does not induce overt toxicity in normal tissues at the tested dose. While the results support its relative safety and tolerability, further studies are necessary to evaluate its pharmacokinetics, conduct comprehensive toxicity profiling, investigate potential immunomodulatory effects, and assess its efficacy across a broader range of cancer models to fully determine its therapeutic potential in BC.

**Figure 9:**
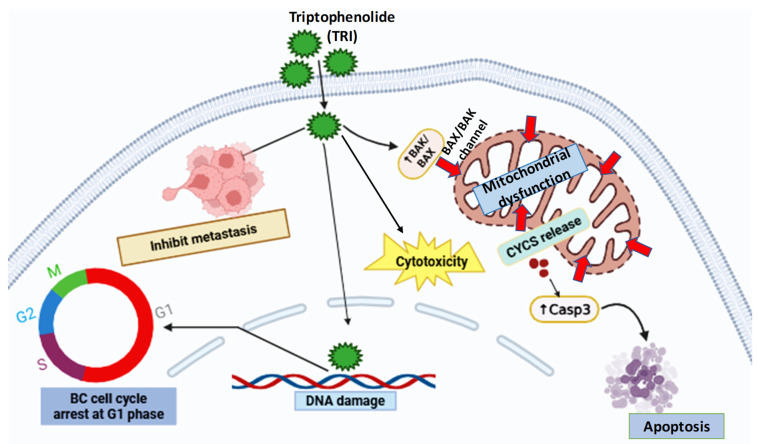
Multi-faceted anti-tumor arsenal of TRI in BC: suppressing proliferation, unleashing apoptosis, halting cell cycle, and blocking metastasis.

Collectively, our integrated analysis reveals TRI’s multi-faceted anti-BC activity, inhibiting migration (90% to 28.5%), reducing xenograft tumor growth, and inducing apoptosis/cell cycle arrest (Figure 9). These results support TRI’s potential as a novel TCM-derived therapeutic. However, future work should aim to identify its precise molecular targets and subtype-specific efficacy. A further limitation of this study is the absence of a standard chemotherapeutic drug as a positive control in both in vitro and in vivo experiments. Including clinically used BC drugs (e.g., doxorubicin or paclitaxel) in future work will enable a more direct comparison of TRI’s efficacy and safety profile relative to current treatment options.

## 4. Materials and Methods

### 4.1. Reagents and Cell Culture

TRI (HPLC > 98.0%, Desite Biotechnology, Chengdu, China) was prepared as a stock solution in dimethyl sulfoxide (DMSO; MP Biomedicals, Strasbourg, France) and stored at −20 °C. BC cell lines (MCF-7 and MDA-MB-231; ATCC) and normal breast cells (MCF-10A; GuangZhou Jennio Biotech Co., Ltd., Guangzhou, China) were maintained in DMEM complete medium (supplemented with 10% fetal bovine serum [FBS; Gibco, Grand Island, NE, USA] and 1% penicillin–streptomycin [PS; Gibco, Grand Island, NE, USA]) at 37 °C in a humidified 5% CO_2_ atmosphere. All cell lines were routinely passaged using 0.25% trypsin-EDTA (Solarbio, Beijing, China) to maintain exponential growth.

### 4.2. Cell Proliferation Assay (CCK-8)

Cells were seeded in 96-well plates at a density of 5 × 10^3^ cells/well (100 μL DMEM medium) and allowed to adhere for 4–6 h. Following attachment, cells were treated with varying concentrations of TRI (0, 100, 200, 300, and 400 μg/mL) in DMEM medium for 24 h or 48 h. At each time point, the medium was replaced with 100 μL fresh DMEM medium containing 10% CCK-8 reagent (Dojindo, Kumamoto, Japan), and plates were incubated for 1.5–2 h at 37 °C. Absorbance was measured at 450 nm using a microplate reader (Thermo Fisher Scientific, Waltham, MA, USA). The half-maximal inhibitory concentration (IC_50_) was calculated using nonlinear regression analysis in GraphPad Prism 8.2.0.

### 4.3. Cell Cycle Analysis

Cells (2 × 10^5^/well) were treated with TN (150 μg/mL) or DMSO (control) for 48 h, harvested, fixed in ice-cold 70% ethanol (−20 °C, overnight), and stained with propidium iodide (RedNucleus I, UE Landy; 4 μL in PBS containing 100 μg/mL RNase A) for 30 min (RT, dark). DNA content was analyzed via flow cytometry (BD FACSVerse; 488 nm laser). A minimum of 10,000 intact nuclei (gated by FSC-A/SSC-A) were recorded per sample. Cell cycle distribution (G0/G1, S, G2/M) was quantified using FlowJo v10.0’s Dean–Jett–Fox algorithm.

### 4.4. Apoptosis Analysis by Annexin V-FITC/PI Dual Staining

Cells (2 × 10^5^ cells/well) were seeded in 6-well plates and treated with either 150 μg/mL TRI (experimental group) or 0.1% DMSO (vehicle control) for 48 h. Following treatment, cells were harvested, washed with cold PBS, and resuspended in 195 μL binding buffer. Cell suspensions were then stained with 5 μL Annexin V-FITC and 10 μL propidium iodide (PI; 20 μg/mL) for 20 min at room temperature in the dark. Apoptotic cells were quantified using a BD FACSVerse flow cytometer (BD Biosciences, Franklin Lakes, NJ, USA) equipped with a 488 nm laser, detecting FITC fluorescence at 530/30 nm and PI emission at 585/40 nm. For each sample, a minimum of 10,000 events were acquired. Data analysis was performed using FlowJo V10 software (BD Biosciences), with viable cell (Annexin V^−^/PI^−^), early apoptotic cell (Annexin V^+^/PI^−^), and late apoptotic/necrotic cell (Annexin V^+^/PI^+^) populations being quantified. The Annexin V-FITC apoptosis detection kit was obtained from Beyotime Biotechnology (Nantong, China).

### 4.5. Cell Migration Assessment by Wound Healing Assay

Cells were seeded in 24-well plates at a density of 2 × 10^5^ cells/well and allowed to adhere overnight. Upon reaching 90–100% confluency, a standardized wound was created in each well using a 200 μL pipette tip. After washing with PBS to remove detached cells, cells were treated with either 150 μg/mL TRI (experimental group) or 0.1% DMSO (vehicle control) in serum-free medium. Wound closure was monitored at 0 h, 12 h, and 24 h using an inverted phase-contrast microscope (ICX41, Ningbo SUNNY Instrument Co., Ningbo, China) with a 10× objective. Three representative images per well were captured at each time point. The migration area was quantified using ImageJ software, version 1.51 j8, by measuring the remaining wound area at each time point relative to the initial wound area (0 h). The migration rate was calculated as follows: Migration rate (%) = [1 − (square area in the visual field for *n* h/square area in the visual field for 0 h)] × 100%. All experiments were performed in triplicate wells with three independent biological replicates.

### 4.6. Quantitative Real-Time PCR (qRT-PCR) Analysis

Cells were treated with either IC_50_ values (180.3 μg/mL and 322.5 μg/mL for MCF-7 and MDA-MB-231 cells) of TRI (experimental group) or 0.1% DMSO (vehicle control) for 48 h. Total RNA was extracted using the RNAprep pure Cell Kit (Tiangen, China), followed by cDNA synthesis using the FastKing RT Kit (Tiangen, China) according to the manufacturer’s protocols. Quantitative PCR was performed using the QuantStudio1 Real-Time PCR System (Thermo Fisher Scientific, Waltham, MA, USA) with PowerUp SYBR Green Master Mix (Thermo Fisher Scientific, Waltham, MA, USA). GAPDH served as the endogenous control for normalization. All primer sequences are listed in Table 1 The thermal cycling conditions followed the manufacturer’s recommendations for the SYBR Green-based detection method. Relative gene expression levels were calculated using the comparative ΔΔCt method, with data representing the mean of three independent biological replicates, each measured in technical triplicate.

### 4.7. Western Blot Analysis

Cells (2 × 10^5^) were treated with IC_50_ values (180.3 μg/mL and 322.5 μg/mL for MCF-7 and MDA-MB-231 cells) of TRI (experimental group) or 0.1% DMSO (vehicle control) for the specified duration. Total protein was extracted using RIPA lysis buffer containing protease inhibitors. Protein concentrations were determined by BCA assay (Thermo Fisher Scientific), with equal amounts (30 μg per lane) separated by 10–12% SDS-PAGE and subsequently transferred to PVDF membranes (0.45 μm; Millipore, Hub Carlsbad, CA, USA). Membranes were blocked with 5% non-fat milk in TBST (Tris-buffered saline with 0.1% Tween-20) for 1 h at room temperature and then incubated overnight at 4 °C with primary antibodies from Cell Signaling Technology (1:1000 dilution in 5% BSA/TBST): rabbit monoclonal antibodies against CYCS, BIM, BAX, and GAPDH (loading control). Following three 10 min TBST washes, membranes were incubated with HRP-conjugated secondary antibodies (goat anti-rabbit IgG or goat anti-mouse IgG, 1:5000; Nakasugi Golden Bridge, Beijing, China) for 1 h at room temperature. Protein bands were visualized using an ECL detection kit (PE0010, Solarbio, Beijing, China) and imaged with a ChemiDoc imaging system (Bio-Rad, Shanghai, China). Band intensities were quantified using ImageJ software (NIH), normalized to GAPDH expression, and expressed as fold-changes relative to control.

### 4.8. Xenograft Tumor Model

Ten female BALB/c nude mice (4 weeks old, 20 ± 2 g; SiPeifu, Beijing China) were maintained under specific pathogen-free conditions. MCF-7 cells (2 × 10^5^ cells in 100 μL PBS) were subcutaneously injected into the right shoulder region of each mouse. When tumors reached approximately 90 mm^3^ (typically 7–10 days post-inoculation), mice were randomly divided into two groups (*n* = 5 per group): (1) the experimental group receiving 10 mg/kg TRI (dissolved in 5% DMSO/PBS, 100 μL; intraperitoneal injection) and (2) the control group receiving vehicle (5% DMSO/PBS, 100 μL; intraperitoneal injection). Treatments were administered every 3 days. Tumor volumes were measured every 3 days using digital calipers and calculated as (length × width^2^)/2. Mice were humanely euthanized when tumors reached 1000 mm^3^ or if signs of distress appeared (per institutional guidelines). Excised tumors were weighed, photographed, and processed for further analysis. Survival rates were recorded and analyzed using Kaplan–Meier curves (GraphPad Prism 8.2.0). TRI was prepared as a 50 mM stock solution in DMSO and stored at −20 °C, with fresh dilutions made for each treatment.

### 4.9. Histopathological Analysis

Tissue samples from both control and TRI-treated mice were collected, fixed in formalin, embedded in paraffin, and sectioned. The sections were then deparaffinized, rehydrated, and stained using hematoxylin and eosin (H&E). After staining, the slides were dehydrated, mounted, and examined under a light microscope for histological evaluation.

### 4.10. Statistical Analysis

All experiments were performed with at least three independent biological replicates, and data are presented as mean ± standard deviation (SD). Statistical analyses were conducted using GraphPad Prism 8.2.0 (GraphPad Software, San Diego, CA, USA). For comparisons between two groups, an unpaired Student’s t-test was applied, whereas one-way or two-way ANOVA (followed by appropriate post hoc tests) was used for multi-group analyses.

Cell migration assays and Western blot band quantification were performed using ImageJ 1.8.0 (NIH, Bethesda, MD, USA; RRID: SCR_003070). Statistical significance was defined as * *p* < 0.05, with asterisks denoting the following: * *p* < 0.05, ** *p* < 0.01, *** *p* < 0.001, and **** *p* < 0.0001.

## 5. Conclusions

In summary, TRI triggered intrinsic apoptosis in BC models through mitochondrial pathway activation (↑BIM, BAK1, BAX, CYCS) and caused DNA damage, while simultaneously inhibiting proliferation and metastasis (migration reduced by 61.5%) (Figure 9). Its in vivo efficacy was confirmed by significant tumor volume reduction and improved survival in xenograft models, with no observable toxicity in major organs based on histopathological evaluation. While these findings support TRI’s potential as a therapeutic candidate, further studies exploring its pharmacokinetics and efficacy across diverse cancer models are warranted to advance its clinical applicability.

## Figures and Tables

**Table 1:** Primer Sequence Information.

Gene	Forward Primer Sequence (5′–3′)	Reverse Primer Sequence (5′–3′)
*GAPDH*	AAGGTGAAGGTCGGAGTCAA	GGAAGATGGTGATGGGATT
*BIM*	CTGAGTGTGACCGAGAAG	GATTACCTTGTGGCTCTGT
*BAK1*	TCTGGCCCTACACGTCTACC	ACAAACTGGCCCAACAGAA
*CASP3*	AAGCGAATCAATGGACTCT	TGTACCAGACCGAGATGT
*CASP9*	CGAACTAACAGGCAAGCA	AATCCTCCAGAACCAATGTC
*BAX*	CCTTTTGCTTCAGGGTTTCA	CAGTTGAAGTTGCCGTCAGA
*TP53*	GTTCCGAGAGCTGAATGAGG	TCTGAGTCAGGCCCTTCTGT
*CYCS*	GGTGATGTTGAGAAAGGCAAG	GTTCTTATTGGCGGCTGTGT
*PIDD1*	TCAGAGGATTCGGACGCAG	GTGAGTGCTCAGACGCAAGAA
*NFKB1*	AACAGAGAGGATTCGTTTCCG	TTTGACCTGAGGGTAAGACTTCT
*AKT1*	TCCTCCTCAAGAATGATGGCA	GTGCGTTCGATGACAGTGG
*CDK4*	CCAGGACCTAAGGACATATC	TGACTGTTCCACCACTTG
*CCND1*	CAATGACCCCGCACGATTTC	CATGGAGGGCGGATTGGAA

## Data Availability

The data that support the findings of this study are available from the corresponding author by e-mail upon reasonable request.
